# The Use of Illegal Drugs and Infectious Contagious Diseases: Knowledge and Intervention among Dockworkers

**DOI:** 10.3390/ijerph13010125

**Published:** 2016-01-12

**Authors:** Marta Regina Cezar-Vaz, Clarice Alves Bonow, Mara Regina Santos da Silva, Francisca Lucélia Ribeiro de Farias, Marlise Capa Verde de Almeida

**Affiliations:** 1 School of Nursing, Federal University of Rio Grande, Rio Grande, RS 96203, Brazil; marare@brturbo.com.br (M.R.S.S.); marlisealmeida@msn.com (M.C.V.A.); 2 Faculty of Nursing, Federal University of Pelotas, Pelotas, RS 96010, Brazil; claricebonow@gmail.com; 3 Graduate Program on Nursing, Fortaleza University, Fortaleza, CE 60811, Brazil; luceliafarias@unifor.br

**Keywords:** occupational health, dockworkers, public health, drugs use

## Abstract

This study’s objective was to analyze the use of illegal drugs by dockworkers and provide risk communication regarding the use of illegal drugs and test for infectious contagious diseases among dockworkers. This cross-sectional study including an intervention addressed to 232 dockworkers, who were individually interviewed, as well as communication of risk with testing for infectious contagious diseases for 93 dockworkers from a city in the interior of Rio Grande do Sul, Brazil. Poisson regression analysis was used. Twenty-nine workers reported the use of illegal drugs. Poisson regression indicated that being a wharfage worker, smoker, having a high income, and heavier workload increases the prevalence of the use of illegal drugs. During risk communication, two workers were diagnosed with hepatitis B (2.2%), three (3.2%) with hepatitis C, two (2.2%) with syphilis. None of the workers, though, had HIV. This study provides evidence that can motivate further research on the topic and also lead to treatment of individuals to improve work safety, productivity, and the health of workers.

## 1. Introduction

The most evident diseases affecting drug users are infectious contagious diseases, such as hepatitis B and C, syphilis and HIV. A systematic review addressing hepatitis B and C in individuals using injectable drugs reports that the prevalence of these diseases is even higher than HIV, which is a public health problem [1]. Even though syphilis presents a high risk of contamination through sexual intercourse, there are also other risk factors such as HIV infection and the use of illegal drugs, such as methamphetamine use [2]. In regard to exposure to HIV, studies address the importance of implementing programs to decrease the risk of infection from the use of injectable drugs, e.g., syringe exchange programs [3,4]. Therefore, it is important to test for these diseases among individuals using illegal drugs.

A second consideration is that drug users pose a risk to individuals who do not use illegal drugs, for instance by working under the effect of drugs. One study testing whether there is a cause-effect relationship between toxicological testing for alcohol and drugs and reduced occupational accidents verified that random and surprise toxicological testing can prevent occupational accidents [5].

The risk posed to people may be even worse when the work performed by individuals using illegal drugs is dangerous, such as the case of port activities. The work performed in ports is globally important because it is in ports where products are received and dispatched to locations around the world [6]. Additionally, as a study previously conducted with the same population shows, the use of drugs in this field of work is frequent, which motivated this study [7].

Individuals using illegal drugs—workers or not—require the attention of healthcare providers because drug users strongly depend on care provided by healthcare services. Such care includes both emergency and primary healthcare. Hence, acquiring more knowledge regarding individuals using illegal drugs helps healthcare providers from all fields to better manage these patients. Specifically for the public health field, dealing with the use of illegal drugs by dockworkers leads to the need for specific knowledge regarding how to manage these situations. This study precedes this management situation; that is, it intends to clarify situations in which illegal drugs are used so that actions are planned jointly with healthcare units in the public health sphere. Therefore, this study’s objective was to analyze the use of illegal drugs among dockworkers, provide risk communication regarding the use of illegal drugs and test these workers for infectious contagious diseases.

## 2. Methods

This cross-sectional study including an intervention used individual interviews to collect data and implemented risk communication regarding the use of illegal drugs, testing dockworkers for infectious contagious diseases (hepatitis B, C, syphilis and HIV) in the interior of the state of Rio Grande do Sul, Brazil.

The port in the interior of the state of Rio Grande do Sul, Brazil, where the study was developed, has 723 dockworkers. The sample was composed after subtracting 25 workers whose work is performed online, 53 workers who were on leave (and not expected to return by the end of data collection); and another 66 workers from specific sectors within the port and therefore not linked to the port labor unions, so that the population totaled 579 dockworkers.

Individual interviews were held with 232 dockworkers. The sample size was selected considering a population of 579 dockworkers and 95% confidence interval. The convenience sample reached the estimated number (*n* = 232). All the dockworkers were invited to participate in the study regardless of the use of drugs.

A structured questionnaire was developed for the interview addressing socio-demographic variables (age, race, marital status and education), occupational variables (occupation in the port facility, monthly income, time working at the port, work shift, mental workload), variables regarding the use of legal drugs (smoking and alcohol), and variables regarding the use of illegal drugs (having any knowledge regarding co-workers using illegal drugs during work and drugs used (amphetamine, marijuana, cocaine, heroin, and ecstasy) [8]. The questionnaire was applied through an interview held in the participants’ workplace in the organized port (maritime) in the interior of the state of Rio Grande do Sul, Brazil.

The National Aeronautics and Space Administration Task Load Index (NASA-TLX) was used to assess mental workload [9]. NASA-TLX measures workload through six scales: mental demand, physical demand, temporal demand, performance, total effort, and frustration. Only the mental demand scale was used in this study because the use of illegal drugs is associated with a high mental task load [10], that is, difficulty in balancing the mental demands of a given task and the mental capacity to perform tasks.

A total of 579 dockworkers were invited to participate in risk communication and to be tested for infectious contagious diseases (hepatitis B, C syphilis and HIV). Fliers were distributed in the workplace to invite the workers. The particular infectious contagious diseases—hepatitis B, C, syphilis and HIV—were chosen because these are reported in the literature as important diseases affecting individuals using illegal drugs [1,11,12]. This stage was jointly implemented by the University, port management, and the City Council of the city in which the port is located in the interior of Rio Grande do Sul, Brazil.

In the data analysis the quantitative variables were described by mean and standard deviation or median and interquartile range. The categorical variables were described by absolute and relative frequency. Student’s *t* test for independent samples was used to compare means between groups, that is, to compare workers who use illegal drugs and those who do not. The Mann-Whitney test was used in the case of asymmetry. Pearson’s Chi-square test or Fisher’s exact test was used to compare proportions and Poisson Regression Analysis was used to control for confounding factors. For a variable to be included in the model, it had to present a *p*-value < 0.20 in the bivariate analysis; and a *p*-value < 0.10 in the final model to remain in the model. The level of significance was set at 5% (*p* ≤ 0.05), and the analyses were conducted using the Statistical Package for Social Science, version 21.0 (IBM SPSS Software, São Paulo, Brazil). All subjects gave their informed consent for inclusion before they participated in the study. The study was conducted in accordance with the Declaration of Helsinki, and the protocol was approved by the Ethics Committee of Federal University of Rio Grande (protocol No. 23116.004481/2013-53).

## 3. Results

The participants who completed the individual interview (232 dockworkers) were mostly Caucasian (*n* = 130; 56%), 141 (60.8%) married, 86 (37.1%) had completed high school, were aged 48.7 years old on average (±10.4), were wharfage workers (*n* = 137; 50.4%), with an average experience of 24.2 years (SD ± 11.3 years), and a monthly income of R$4248.76 (SD ± R$2429.27) (Table 1).

**Table 1:** Sociodemographic and occupational characteristics of dockworkers in the interior of the state of Rio Grande do Sul, Brazil.

Variables	*n*	%
Race		
Caucasian	130	47.8
Afro-Descendant	54	19.9
Mixed	34	12.5
Asian	08	2.9
Indigenous	06	2.2
Marital Status		
Married	141	51.8
Single	49	18.0
Separated/Divorced	35	12.9
Widowed	07	2.6
Education *		
Incomplete primary or middle school	67	24.6
Complete middle school	35	12.9
Incomplete high school	22	8.1
Complete high school	86	31.6
Some college study	10	3.7
Bachelor’s degree or a graduate degree	09	3.3
Professional Occupation		
Wharfage worker	137	50.4
Longshoreman	78	28.7
Cargo checking	17	6.3

* Three dockworkers (1.1%) reported being illiterate.

All the dockworkers were asked whether their co-workers had ever worked under the effect of illegal drugs and most 212 (91.4%), reported being aware of dockworkers working under the effect of illegal drugs. These individuals reported the following illegal drugs were used by co-workers: 147 (63.4%) reported marijuana, 124 (53.4%) reported cocaine, and crack cocaine was reported by 95 (40.9%) workers. The reasons for which they believe their co-workers resort to illegal drugs included: dependence, reported by 121 (52.2%) workers; 24 (10.3%) mentioned to decrease tiredness; and 14 (6.0%) said co-workers used drugs to gain courage to perform their tasks. In regard to legal drugs, 59 (25.4%) workers reported the use of cigarettes and 119 (51.3%) reported the use of alcohol.

Of the total number of dockworkers interviewed (*n* = 232), 29 (12.5%) reported personal use of illegal drugs, namely marijuana was reported by 27 (93.1%) workers; cocaine was reported by six (20.7%); one (3.4%) worker reported the use of crack; inhalant solvent was reported by one (3.4%); and another worker (3.4%) reported the use of illegal drugs but did not specify which ones. The workers who reported the use of illegal drugs justified their use due to dependence (13; 44.8%); to decrease tiredness (3; 10.3%); and one (3.4%) reported it helped give courage to perform tasks.

Significant association was found in the bivariate analysis regarding the use of illegal drugs with smoking (*p* = 0.019), working hours (*p* = 0.015) and the mental demand accruing from working in the port (*p* = 0.023). In the association of occupation and the use of illegal drugs, a significantly higher prevalence of illegal drugs was found among longshoremen (*p* = 0.021) and wharfage workers (*p* = 0.037) (Table 2).

**Table 2:** Bivariate analysis of illegal drugs with sociodemographic, occupational and personal variables of dockworkers in the interior of Rio Grande do Sul, Brazil.

Variables *	User of Illegal Drugs (*n* = 29)	Non-User of Illegal Drugs (*n* = 203)	*p-*Value
Age group	45.7 ± 10.2	49.2 ± 10.4	0.088
			0.393
<40 years old	9 (31.0)	41 (20.2)	
From 40 to 59 years old	16 (55.2)	124 (61.1)	
≥60 years old	4 (13.8)	38 (18.7)	
Education			0.384
Illiterate/Incomplete elementary	8 (27.6)	62 (30.5)	
Complete middle school	2 (6.9)	33 (16.3)	
Incomplete high school	1 (3.4)	21 (10.3)	
Complete high school	15 (51.7)	71 (35.0)	
Some college studies	2 (6.9)	8 (3.9)	
Bachelor’s or a graduate degree	1 (3.4)	8 (3.9)	
Monthly income (R$)	4000 (3000–5000)	3600 (2733–5000)	0.199
Professional occupation			
Wharfage	17 (7.3)	120 (51.7)	0.037
Longshoreman	12 (5.1)	66 (28.4)	0.021
Cargo checking	0 (0.0)	17 (7.3)	0.285
Time working in the port (years)	22.5 ± 10.4	24.5 ± 11.5	0.380
Working hours	8.48 ± 3.01	6.98 ± 2.29	0.015
Robust variable (hours **x** years)	144 (120–207)	150 (120–210)	0.899
Work shift			0.532
Only day shift	2 (6.9)	30 (14.8)	
Only night shift	1 (3.4)	15 (7.4)	
Night and Day shifts	25 (86.2)	153 (75.4)	
Other	1 (3.4)	5 (2.5)	
Mental task load	15.0 ± 4.3	12.7 ± 5.1	0.023
Smoker	13 (44.8)	46 (22.7)	0.019
Alcohol use	18 (62.1)	101 (49.8)	0.297

* variables described by mean ± standard deviation, median (percentiles 25–75) or n (%).

Poisson regression showed that wharfage workers presented prevalence 123% higher for the use of illegal drugs and smokers presented a prevalence 160% higher for illegal drugs than non-smokers. Income and working hours were also associated with the use of illegal drugs: an increase of R$100 increases by 1% the likelihood of using illegal drugs, while one extra hour of dock work increases the likelihood of using illegal drugs by 17% (Table 3).

**Table 3:** Occupational and personal factors associated with increased use of illegal drugs among dockworkers in the interior of Rio Grande do Sul, RS, Brazil.

Variables	PR (CI 95%) *	*p-*value
Wharfage work	2.23 (1.18–4.22)	0.014
Smoking	2.60 (1.37–4.93)	0.003
Income (x100)	1.01 (1.00–1.03)	0.041
Working hours	1.17 (1.05–1.29)	0.003

* Ratio of prevalence with confidence interval of 95%.

**Figure 1:**
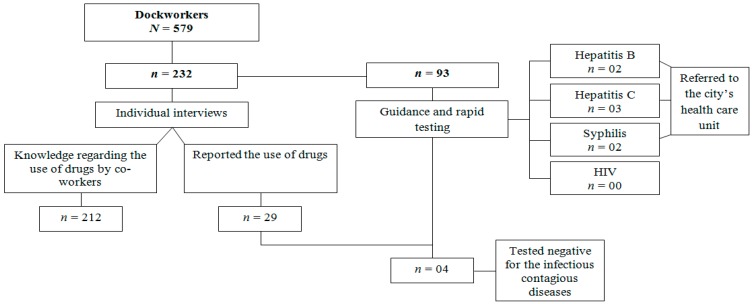
Scheme of the results.

Five meetings were conducted to implement risk communication and provide counseling regarding the risk of illegal drugs and rapid tests to diagnose infectious contagious diseases—Hepatitis B, C, syphilis and HIV. The meetings were held between October 2014 and March 2015. All the meetings were conducted in a room previously arranged by the port management on the premises of the participants’ workplace. Each meeting gathered 20 workers, on average, totaling 93 workers (16.6% of the population). Only four of those reporting the use of illegal drugs participated in risk communication, though they presented no infectious contagious diseases. In regard to the rapid tests, two (2.2%) workers tested positive for hepatitis B, three (3.2%) tested positive for hepatitis C, two (2.2%) for syphilis, while no participants tested positive for HIV. All the participants who tested positive were referred to the city’s testing and counseling center. Figure 1 presents a synthesis of the results.

## 4. Discussion

Illegal drugs were frequently used by dockworkers as most participants, 212 (91.4%), reported co-workers work under the effect of drugs. This behavior poses a risk to other workers, since the activities performed in the docks are collective. It is known that implementing random and surprise toxicological testing at the workplace decreases the risk of the use of illegal drugs among workers, which consequently has the potential to decrease the exposure of workers to occupational accidents [5]. Nonetheless, the implementation of toxicological testing in Brazil is seldom discussed and a controversial issue. The reason is that according to Brazilian law, workers are not obliged to undergo this type of testing. Brazilian law No. 13.103 from 2 March 2015 [13], determines that toxicological tests can be demanded prior to hiring and at the time of exit or resignation only for professional drivers. Therefore, in Brazil, the worker decides whether to undergo toxicological testing at the workplace so that a worker undergoes the test only if she/he wants to. As a consequence, the test can no longer be a surprise. In Italy, the implementation of such testing is different, as the law of drug testing at the workplace was enacted in 2008. A study intended to analyze data found in drug testing at the workplace shows the results did not change over time, that is, workers do not decrease the use of drugs because they were being tested at work [14].

It is worth noting that the workers who reported being aware that co-workers worked under the influence of drugs, did so voluntarily. This shows these workers hold no prejudice against the use of illegal drugs; on the contrary, they acknowledge it as a common habit among dockworkers. Additionally, since the interviews were conducted in the participants’ workplace, the team responsible for collecting data was able to observe on various occasions workers making use of illegal drugs. Another situation observed during the interviews was one worker covering up for another worker using illegal drugs. Because port activities are performed as block work [15], that is, groups of workers who become responsible for a given daily task, the workers choose what activity and the group with which they will work. Because workers who do not use illegal drugs know the ones who do use them, there were situations in which workers chose a group in which there was a drug user in order to protect him and also situations in which one worker warned another not to chose a given group because there was a worker who was a user of illegal drugs.

Even though a large number of workers reported they knew individuals who worked under the influence of drugs, few workers (*n* = 29) reported the use of illegal drugs. Underreporting may be caused by embarrassment, on the part of these workers, of admitting the use of drugs or fear of being reported to authorities since the use of drugs is illegal. A study addressing the use of drugs among steel mill workers shows that self-report was the least effective (comparing to chemical analysis of urine and hair) to estimate the prevalence of drugs use [16]. It agrees with the conclusion of a study conducted with cocaine users that shows that self-reporting of drugs use may require confirmation through biochemical analysis [17]. Therefore, this study presents a limitation concerning the identification of dockworkers using illegal drugs. Self-reporting is, however, the first step toward the identification of characteristics that should be studied in certain environments. We suggest further studies to improve the identification of other workers using illegal drugs and to implement new interventions.

The drugs used by the workers include marijuana, cocaine, crack and inhaling solvents. Marijuana, the illegal drug most frequently reported by the dockworkers, is identified in the literature as a drug that can be mixed in use with others, e.g., cocaine [18]. Additionally, it is known that the use of an illegal drug increases the likelihood that other drugs be adopted [19], even if they were not reported by the dockworkers.

When the workers were asked about the reasons that led their co-workers to use illegal drugs, the one most frequently reported was dependence. It is known that the use of illegal drugs causes chemical dependence, that is, an organic condition initiated with the use of psychoactive drugs that lead to the onset of symptoms such as an uncontrollable desire for the drug, loss of control, and increased intolerance [20]. This is an important clinical condition and, for this reason, healthcare workers should be attentive. The International Classification of Diseases [21] presents a block—Mental and behavioural disorders due to psychoactive substance use (F10–F19)—that contains a variety of diseases attributed to the use of psychoactive substances, showing the importance of identifying the use of illegal drugs and the chemical dependence it causes. The proposal that it decreases tiredness was also used, by the drug users and their co-workers, to justify the use of drugs. This effect is mainly associated with cocaine, because it decreases tiredness and is used as a stimulant [22].

Another reason reported by the participants for their co-workers’ use of drugs was that it gives them courage to perform their tasks. Port activities are dangerous [23] and this explains the workers’ need to gain courage; however, this is not the best way to perform a task. They justify their use of drugs to acquire courage to perform the tasks but doing so jeopardizes their safety and that of their co-workers. In addition to causing euphoria, cocaine intensifies one’s sense of confidence [18]. This situation is well-suited to port work because this substance enables workers to become more courageous, decreasing the fear of heights and of falling into the sea, making them work faster increasing their productivity.

The results of Poisson regression analysis indicate that wharfage workers, those with higher incomes, longer working hours, and smokers present a higher prevalence of illegal drug use. The function of wharfage workers is to move the goods within the port facilities, being responsible for receiving, checking, internal transportation, opening packages for custom inspection, handling, storage, delivery, loading and unloading vessels [15]. There are various types of products wharfage workers have to move: containers (with clothing, meat, computers), liquid products (fuel and vegetable oils), solid products (grains, charcoal, cement), fractionated products (paper, wood, steel, wind turbines) and roll-on/roll-off products (cars, buses, trucks, agricultural vehicles, mounted cranes) [24]. These maneuvers require physical strength and even though this study does not more deeply analyze the labor issues that lead individuals to use illegal drugs, it is acknowledged that wharfage workers perform many tasks that require physical strength, which may contribute to the use of illegal drugs, as some drugs such as cocaine cause euphoria [18]. Hence, higher income and longer working hours are associated with the use of illegal drugs because, as previously mentioned, some drugs function as stimulants [22].

In regard to the increased use of illegal drugs among smokers, it is known that individuals who use illegal drugs present high rates of smoking. A study that sought smoking patterns among illegal drug users shows that most drug users also smoke cigarettes [25]. This result presents directions for healthcare professionals also to address the use of tobacco when treating dependence to illegal drugs.

During risk communication, we also identified individuals with infectious contagious diseases (hepatitis B, C and syphilis). Hepatitis B and C are frequently mentioned in the literature as diseases related to the use of illegal drugs [11,26], which are even more frequent than HIV [1]. A 33-year retrospective cohort study examined the monitoring of causes of mortality among injectable drug users affected by hepatitis C. The analysis indicated that mortality among drug users is more frequently linked to the use of injectable drugs (intoxication, suicide) than to complications accruing from hepatitis C [11]. Therefore, it is important to diagnose and refer patients to specialized services to initiate the treatment for hepatitis and also to refer the user to a rehabilitation service. The diagnosis of syphilis is of concern because this is an infectious contagious disease that affects pregnant women and leads to congenital syphilis, that is, it is a disease that may be transmitted to infants and lead to severe complications. The World Health Organization estimates 1 million cases of syphilis every year among pregnant women [27]. Even though pregnant women were not the focus of this study, the concern here is to provide timely treatment for their sexual partners affected by syphilis.

Only four of the dockworkers who reported the use of illegal drugs during the individual interviews participated in risk communication addressing illegal drugs. This low adherence from illegal drug users shows that individual interventions need to be implemented among those using illegal drugs, since these individuals did not accept the collective invitation. In this sense, we recommend deepening the topic regarding the use of drugs among dockworkers and other workers by identifying frequency and quantity of illegal drug use, as well as occupational accidents for which these individuals may be responsible. For this reason, workers using illegal drugs require more attention at work than those who do not use drugs. The fact that these individuals use drugs jeopardizes their safety and that of those workers who do not use illegal drugs, as will be reflected in the collective nature of port work and activities.

## 5. Conclusions

The conclusion is that there is a need to direct attention to the use of illegal drugs among dockworkers and others performing collective tasks, such as professional drivers, construction workers and police, that is, activities in which safety depends not on a single individual but on co-workers. Hence, this study provides evidence that motivates future research on the topic seeking the treatment of these individuals to improve occupational safety, productivity and occupational health.
